# Setup accuracy and margins for surface-guided radiotherapy (SGRT) of head, thorax, abdomen, and pelvic target volumes

**DOI:** 10.1038/s41598-023-44320-2

**Published:** 2023-10-09

**Authors:** Volker Rudat, Yanyan Shi, Ruping Zhao, Shuyin Xu, Wei Yu

**Affiliations:** Department of Radiation Oncology, Jiahui International Cancer Center Shanghai, Jiahui Health, Shanghai, China

**Keywords:** Cancer, Medical research, Oncology

## Abstract

The goal of the study was to evaluate the inter- and intrafractional patient setup accuracy of target volumes located in the head, thoracic, abdominal, and pelvic regions when using SGRT, by comparing it with that of laser alignment using patient skin marks, and to calculate the corresponding setup margins. A total of 2303 radiotherapy fractions of 183 patients were analyzed. All patients received daily kilovoltage cone-beam computed tomography scans (kV-CBCT) for online verification. From November 2019 until September 2020, patient setup was performed using laser alignment with patient skin marks, and since October 2020, using SGRT. The setup accuracy was measured by the six degrees of freedom (6DOF) corrections based on the kV-CBCT. The corresponding setup margins were calculated using the van Herk formula. Analysis of variance (ANOVA) was used to evaluate the impact of multiple factors on the setup accuracy. The inter-fractional patient setup accuracy was significantly better using SGRT compared to laser alignment with skin marks. The mean three-dimensional vector of the translational setup deviation of tumors located in the thorax, abdomen, and pelvis using SGRT was 3.6 mm (95% confidence interval (CI) 3.3 mm to 3.9 mm) and 4.5 mm using laser alignment with skin marks (95% CI 3.9 mm to 5.2 mm; *p* = 0.001). Calculation of setup margins for the combined inter- and intra-fractional setup error revealed similar setup margins using SGRT and kV-CBCT once a week compared to laser alignment with skin marks and kV-CBCT every other day. Furthermore, comparable setup margins were found for open-face thermoplastic masks with AlignRT compared to closed-face thermoplastic masks with laser alignment and mask marks. SGRT opens the possibility to reduce the number of CBCTs while maintaining sufficient setup accuracy. The advantage is a reduction of imaging dose and overall treatment time. Open-face thermoplastic masks may be used instead of closed-face thermoplastic masks to increase the patient's comfort.

## Introduction

Technological advances in radiotherapy like volumetric-modulated arc radiotherapy (VMAT) have enabled the delivery of highly conformal dose distributions in routine radiotherapy^1^. Accurate patient positioning is critical to correctly deliver the planned dose distribution. The increasing use of hypofractionated protocols and stereotactic body radiotherapy (SBRT) enhances the importance of the patient positioning accuracy. The patient setup in routine radiotherapy is usually performed by alignment of in-room lasers with patient skin marks and the verification of the patient setup by cone-beam computed tomography (CBCT). CBCT should be performed daily to achieve optimal patient positioning accuracy with minimal setup margins. However, daily online verification using CBCT increases the dose to normal tissue and the overall treatment time resulting in a lower number of patients that can be treated per day. A lower frequency of online verifications requires larger setup margins to compensate for the inter-fractional patient setup error, and larger target volumes increase the risk of radiation toxicity. A possible strategy to reduce the number of CBCTs while maintaining sufficient patient positioning accuracy could be the use of surface guided radiotherapy (SGRT).

SGRT is a technology that uses an optical surface monitoring system (OSMS) for patient positioning, intra-fraction motion monitoring and respiratory gating^2^. A real-time three-dimensional (3D) surface of the patient is generated by a combination of projectors and cameras. The patient’s real-time surface is then compared to a reference surface to derive shifts in six degrees of freedom (6DOF). An automated beam hold can be performed if the difference between surfaces exceeds a predefined threshold.

SGRT offers several advantages compared to image guided radiotherapy (IGRT) using orthogonal X-ray imaging, portal imaging or CBCT. Firstly, SGRT does not add radiation dose to the patient and therefore may contribute to the desired imaging dose reduction. Secondly, SGRT allows monitoring of the patient position during treatment thus reducing intra-fractional error. Although X-ray based IGRT using triggered imaging is available, it does not provide continuous monitoring compared to SGRT. In addition, SGRT allows assessing and monitoring the patient position in non-coplanar couch angles. Finally, patient setup and overall treatment time is shorter with SGRT compared to CBCT^3^.

A popular application of SGRT is the radiotherapy of left breast cancer using deep inspiration breath-hold (DIBH) technique^4–6^. Another example of SGRT application is the treatment of brain or head and neck tumors with open-face mask^7–9^. However, the value of SGRT for tumors not located close to the skin surface has not yet been clearly defined.

In our retrospective study of patients with target volumes of the head and neck, thorax, abdomen, and pelvis, we compared the setup accuracy of patients positioned using SGRT with patients positioned using in-room laser alignment with patient skin marks. All patients received daily kilo-voltage cone-beam computed tomography (kV-CBCT) for online setup verification, with positioning accuracy represented by the setup correction based on kV-CBCT. Population systematic and random errors were calculated^10^ and the setup margins to compensate for the inter- and intra-fractional error estimated using the van Herk formula^11^. From November 2019 until September 2020, the patient positioning was achieved by aligning in-room lasers with patient skin marks. Since October 2020, patients were positioned using OSMS. During the first irradiation fraction, the patient was positioned using laser alignment with skin markers. SGRT has the potential to improve patient setup compared to laser alignment to skin marks with the advantage of potentially reducing the inter- and intra-fractional patient positioning error. In patients where clinically appropriate, the increased patient setup accuracy may allow a reduction in the number of CBCTs, thereby reducing the imaging dose and overall treatment time. In addition, the setup accuracy of open-face masks using SGRT was compared to that of closed-face masks using laser alignment. If the setup accuracy is comparable, open-face masks would be an alternative for patients who cannot tolerate closed-face masks.

## Methods

###  Ethics approval and consent to participate

The present study was approved by the Institutional Review Board (IRB) of the Jiahui International Hospital, Shanghai, China. Due to the retrospective nature of the study the IRB waived the need of obtaining informed consent (reference number A-JIHSCRJICC2022002-01). The study was conducted in accordance with the ethical guidelines of the Declaration of Helsinki. All methods were executed in accordance with the relevant guidelines and regulations of the captioned IRB.

### Data collection

Patient and treatment related data of patients treated between September 2019 and November 2020 were obtained from the hospital information system (HIS) and the integrated oncology management system MOSAIQ (Elekta AB, Stockholm, Sweden). The data were transferred into a custom-made database (Access, Microsoft, Redmont, USA). After completion of the data collection, data were anonymized and transferred into a statistical software program for analysis (Statistica, TIBCO Software Inc., 2020. Data Science Workbench, version 14. http://tibco.com).

### Pretreatment workflow

For fixation of the head, closed-face thermoplastic masks or open-face thermoplastic masks were used. Positioning devices used for the thorax were breast board, wing board and knee fix, for the abdomen and pelvis, a knee fix and feet fix. Patients receiving SBRT were positioned using a wing board. All patients were treated in supine position without the use of vacuum bags. All positioning devices were obtained from CIVCO Medical Instruments (CIVCO Medical Instruments Co Inc. Orange, IA, USA). CT-simulation was performed using a Brilliance Big Bore CT (Philips, Amsterdam, Netherlands). CT scans were routinely acquired with 3 mm slice thickness and 2 mm for SBRT cases. All patients received skin marks by means of conventional 3-point localization. Target volumes were contoured according to the corresponding RTOG atlas^12^ and NCCN Clinical Practice Guidelines in Oncology (NCCN Guidelines®)^13^. Automatic contouring of organs at risk (OAR) was performed using AccuContour Version 3.1 (Manteia Technology LTD, Xiamen, China). The treatment planning system was Monaco Version 5.40.03 (Elekta AB, Stockholm, Sweden). Radiotherapy was delivered using VersaHD (Elekta AB, Stockholm, Sweden), with Agility MLC (5 mm leaves) and a Hexapod 6D treatment couch. Daily online verification was performed using kV-CBCT and translational and rotational errors corrected by adjusting the Hexapod 6D treatment couch. No abdominal compression or respiratory gating was used for the SBRT of lung or liver targets.

### SGRT

The SGRT system AlignRT (Vision RT Ltd, London, UK, Version 5.1.2) was used for this study. AlignRT saves the patient’s real-time delta data during treatment in the logfile. Real-time delta refers to the deviation in 6 DOF delta between patient’s real-time surface and the reference surface imported from CT or captured by AlignRT. The delta values calculated are based on the registration of the regions of interest (ROI) defined by the Radiographer on the reference surface. For patients with an open-face mask fixation, the region of interest (ROI) was the visible face; for target volumes of the chest, the chest without the supraclavicular or axillary region; for breast patients, the ipsilateral breast and sternum; for the abdomen, the central and lateral abdominal area; and for the pelvis, the pelvis, avoiding deformable structures like the gastric region. The resolution of the AlignRT camera was 2048 × 1024 pixel. A rigid registration algorithm was used to compute the deviation between the patient’s real-time surface and the reference surface. Translational and rotational deviations were corrected by adjusting the 6DOF treatment couch. For the analysis of the intra-fractional setup error, the real-time data acquired during the beam delivery were obtained from the AlignRT log file and transferred into a statistical software program for analysis.Only data collected during the beam on time were evaluated. Data representing extreme outliers were considered measurement errors and were omitted from the analysis. Extreme outliers were defined as data beyond the bounds of the first quartile minus 1.5 interquartile ranges or the third quartile plus 1.5.

### OSMS workflow

**Figure 1:**
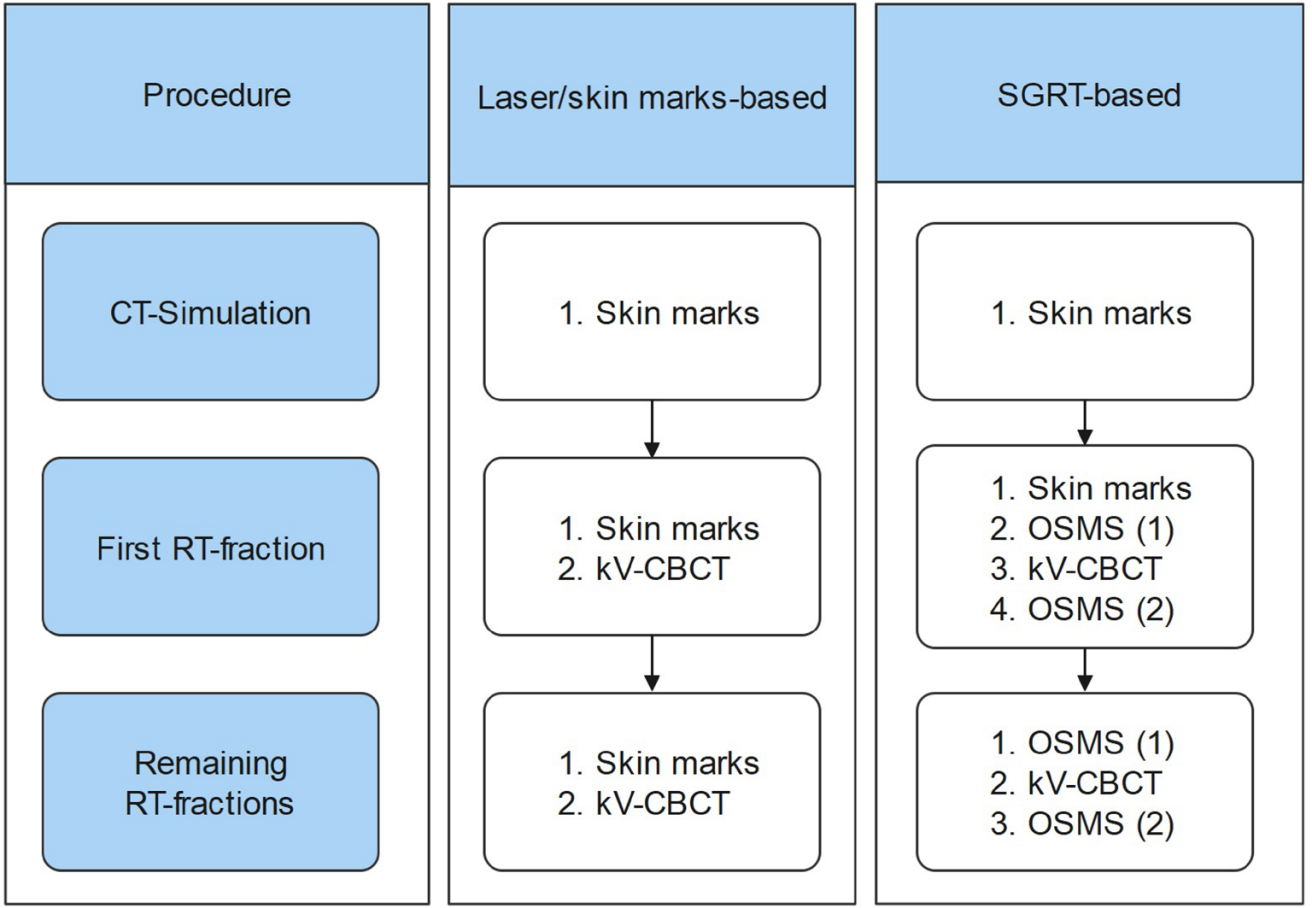
Workflow of the patient setup procedure. OSMS (1) denotes a surface scanning image to be matched with reference surface image from the CT-simulation, and OSMS (2) a surface scanning image as reference to assess the intra-fractional error.

Using OSMS, the patient’s real-time surface was positioned to match reference surface from the CT-simulation. A kV-CBCT was performed, and the patient setup corrected by adjusting the 6DOF treatment couch. A new reference surface was acquired using OSMS to evaluate the intra-fractional setup error. In cases where translational setup deviations exceeded 5 mm or rotational errors 3° (2° for patients with open-mask fixation), the beam was stopped manually, and the patient setup corrected. No automated beam hold was activated because the gantry motion had to be observed carefully anyway due to the possibility of blocking the field of view of the SGRT cameras. For the remaining radiotherapy fractions, the procedure of laser alignment with skin marks was omitted (Fig. 1). In a small number of patients who required more time to set up with OSMS, laser alignment with skin markers was used prior to OSMS to reduce patient setup time.

All patients using open-face thermoplastic masks were treated using OSMS. For patients using closed-face thermoplastic, OSMS was not used. Patients treated using DIBH (usually left-sided breast cancer patients) were excluded from the analysis as the patient setup procedure was different compared to treatment without DIBH.

### Statistical analysis

#### Inter-fractional setup error

An analysis of variance (ANOVA) was performed to assess the impact of the patient setup technique (skin marks vs. SGRT), treatment location (thorax vs. abdomen vs. pelvis), gender (female vs. male), age (≤ mean vs. > mean), weight (≤ mean vs. > mean), BMI (≤ mean vs. > mean), and fractionation regimen (conventional fractionation vs. hypofractionation vs. SBRT) on the inter-fractional setup accuracy. The inter-fractional setup accuracy was represented by the mean three-dimensional vector of the patient setup deviation in the lateral, longitudinal and vertical direction. A two-sided *p*-value ≤ 0.05 was considered statistically significant.

#### Intra-fractional setup error

The impact the location of the target volume (thorax vs. abdomen vs. pelvis), fractionation regimen (conventional fractionation vs. hypofractionation vs. SBRT), gender (female vs. male), age (≤ mean vs. > mean), weight (≤ mean vs. > mean) and BMI (≤ mean vs > mean) on the three-dimensional vector of the intra-fractional setup error was assessed using an ANOVA. A two-sided *p*-value ≤ 0.05 was considered statistically significant.

#### Calculated safety margins

The patient setup accuracy was assessed by calculating the overall population mean setup error (M_pop)_), population systematic (Σ_setup_) and population random error (σ_setup_) of the translational and rotational errors in three directions. The calculations were performed according to the report "On target: ensuring geometry accuracy in radiotherapy" by the Royal College of Radiologists^10^. The patient setup parameters were calculated for each direction (lateral, longitudinal, and vertical). Treatment margins to compensate for the patient setup error were estimated using the van Herk formula^11^. To evaluate the impact of the frequency of online verifications (once per week or every other day) on the calculated setup margin, the patient setup parameters were calculated assuming a patient setup error of 0 mm in all directions after online verification using kV-CBCT.

## Results

### Patient demographics, treatment regime and parameters

A total of 2303 CBCTs of 199 target volumes from 183 patients were analyzed. Of these, 14 patients had more than one tumor located in different anatomical regions and treated in different radiotherapy series. Table 1 shows patient and treatment related characteristics of patients positioned based on skin marks or OSMS.

**Table 1:** Patient and treatment related characteristics.

Error type	Interfractional error				Intrafractional error	
Setup type	Skin marks		OSMS		OSMS	
	n	%	n	%	n	%
Number of radiotherapy fractions	745	–	1558	–	819	–
Total number of target volumes	59	–	140	–	69	–
Number of patients	51	–	132	–	69	–
Anatomical region of target volumes (number of target volumes (number of patients))						
Head and Neck (closed mask)	13 (13)	22.0	–	–	–	–
Head and Neck (open mask)	–	–	31 (31)	22.1	13	18.8
Thorax	23 (22)	39.0	41 (41)	29.3	20	29.0
Abdomen	16 (14)	27.1	55 (53)	39.3	26	37.7
Pelvis	7 (7)	11.9	13 (13)	9.3	10	14.5
Fractionation regimen (number of target volumes (number of patients))						
Conventional fractionation	21 (21)	35.6	23 (23)	16.4	10	14.5
Hypofractionation	32 (26)	54.2	96 (93)	68.6	52	75.4
SBRT	6 (5)	10.2	21 (20)	15.0	7	10.1
Gender (number of target volumes (number of patients))						
Female	26 (25)	44.1	67 (64)	47.9	38	55.1
Male	33 (26)	55.9	73 (68)	52.1	31	44.9
Age						
Mean (SD)	56	(17)	55	(13)	54	(12)
Weight						
Mean (SD)	64	(13)	64	(14)	62	(12)
BMI						
Mean (SD)	23	(4)	23	(4)	22	(3)

*SGRT* Surface guided radiotherapy; *SBRT* Stereotactic body radiotherapy; *BMI* Body mass index; *SD* Standard deviation.

Target volumes were located in the head and neck (n = 44), thorax (n = 64), abdomen (n = 71), and pelvis (n = 20). The most common diagnosis of target volumes in the head and neck was brain metastasis and glioblastoma multiforme (n = 26), of the thorax breast cancer (n = 37), of the abdomen hepatobiliary cancer (n = 45) and of the pelvis cervical cancer (n = 8). The most common conventional fractionation regimen consisted of 30 fractions, hypofractionated regimen of 15 fractions and SBRT of five fractions.

### Inter-fractional setup error

The ANOVA revealed the patient setup technique as the only significant impact factor on the inter-fractional setup accuracy (OSMS: mean 3.6 mm, 95% confidence interval (CI) 3.3 mm to 3.9 mm; laser alignment with skin marks: 4.5 mm, 95% CI 3.9 mm to 5.2 mm; *p* = 0.001).

**Figure 2:**
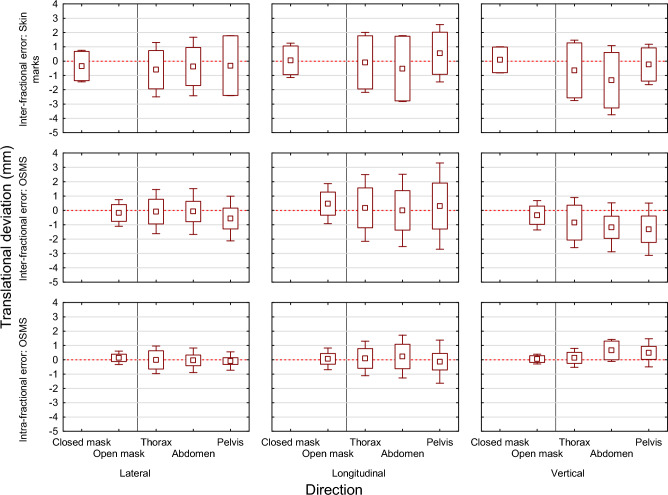
Overall population mean setup error (rectangle), population systematic error (box), and population random error (whiskers) of the inter-fractional translational error using laser alignment with skin marks, inter-fractional translational error using OSMS, and intra-fractional translational error (mm) using OSMS by direction and location of the target volume.

Figure 2 and Table 2 demonstrate that patients of the inter-fractional setup error using OSMS showed smaller population systematic errors compared to the inter-fractional error using laser alignment with skin marks on average by a factor of 1.6. The population systematic error is the most important factor because it has the largest impact on the calculated setup margin to compensate for the patient setup error^11^. Furthermore, Fig. 2 suggests that in the vertical direction there may be an inter-fractional population systematic setup error of 1 mm. A sagging of the treatment table was excluded by phantom measurements and no laser misalignment was detected. The reason for this deviation is yet unclear. The Student’s t-test in Table 2 shows significant differences in the inter-fractional setup accuracy between patients positioned using skin marks versus OSMS for thoracic and abdominal target volumes. For pelvic target volumes, no significant difference was observed. This may be due to the comparatively low number of patients with pelvic target volumes. As expected, patients with head fixation showed smaller inter- and intra-fractional population systematic and random errors compared to those treated in the thorax, abdomen, and pelvis (on average by a factor of 1.5). Fixation with open-face thermoplastic masks show a similar inter-fractional setup accuracy compared to closed-face thermoplastic masks (Table 2).

**Table 2:** Patient setup error in dependence of the setup procedure for each scenario.

Setup type			Skin marks									OSMS									
Direction			LAT			LNG			VRT			LAT			LNG			VRT			*p*-value
Setup error			M	Σ	σ	M	Σ	σ	M	Σ	σ	M	Σ	σ	M	Σ	σ	M	Σ	σ	
Region	Error type	Number of online verifications using CBCT																			
Head and neck (closed mask)	Interfractional	None	− 0.3	1.0	1.1	0.1	1.0	1.2	0.1	0.9	0.9	–	–	–	–	–	–	–	–	–	–
Head and neck (open mask)	Interfractional	None	–	–	–	–	–	–	–	–	–	− 0.2	0.6	0.9	0.5	0.8	1.4	− 0.3	0.6	1.0	–
Thorax	Interfractional	None	− 0.6	1.3	1.9	− 0.1	1.9	2.1	− 0.6	2.1	1.9	− 0.1	0.9	1.5	0.2	1.4	2.3	− 0.8	1.2	1.8	0.03
Abdomen	Interfractional	None	− 0.4	1.3	2.0	− 0.5	2.3	2.3	− 1.3	2.4	1.9	− 0.1	0.7	1.6	0.0	1.4	2.5	− 1.2	0.8	1.7	0.01
Pelvis	Interfractional	None	− 0.3	2.1	2.1	0.6	2.0	1.5	− 0.2	1.2	1.4	− 0.6	0.7	1.6	0.3	1.6	3.0	− 1.3	0.9	1.8	0.78
Head and neck (closed mask)	Interfractional	Once per week	− 0.3	0.8	1.0	0.0	0.8	1.1	0.1	0.7	0.9	–	–	–	–	–	–	–	–	–	–
Head and neck (open mask)	Interfractional	Once per week	–	–	–	–	–	–	–	–	–	− 0.1	0.5	0.8	0.3	0.6	1.2	− 0.2	0.5	0.9	–
Thorax	Interfractional	Once per week	− 0.4	1.1	1.8	− 0.1	1.6	2.0	− 0.5	1.6	1.8	0.0	0.7	1.4	0.2	1.2	2.0	− 0.8	1.0	1.5	0.02
Abdomen	Interfractional	Once per week	− 0.3	1.0	1.8	− 0.4	1.6	2.3	− 1.0	1.6	2.1	− 0.1	0.6	1.3	− 0.1	1.1	2.3	− 1.0	0.7	1.6	0.03
Pelvis	Interfractional	Once per week	− 0.2	1.3	2.1	0.4	1.5	1.7	− 0.2	1.0	1.4	− 0.4	0.7	1.4	0.1	1.3	2.8	− 1.1	0.7	1.7	0.42
Head and neck (closed mask)	Interfractional	Every other day	− 0.1	0.4	0.8	0.1	0.5	0.9	0.1	0.4	0.7	–	–	–	–	–	–	–	–	–	–
Head and neck (open mask)	Interfractional	Every other day	–	–	–	–	–	–	–	–	–	− 0.1	0.4	0.7	0.2	0.4	0.9	− 0.1	0.4	0.8	–
Thorax	Interfractional	Every other day	− 0.3	0.7	1.4	0.0	1.1	1.6	− 0.3	1.1	1.6	0.0	0.5	1.1	0.2	0.8	1.5	− 0.4	0.6	1.3	0.02
Abdomen	Interfractional	Every other day	− 0.2	0.6	1.5	− 0.1	1.0	1.9	− 0.6	0.9	1.7	− 0.1	0.6	1.1	− 0.2	0.9	1.8	− 0.5	0.5	1.3	0.03
Pelvis	Interfractional	Every other day	− 0.1	1.4	2.1	0.5	1.0	1.5	− 0.3	0.4	1.0	− 0.3	0.4	1.1	0.0	0.6	1.9	− 0.5	0.5	1.4	0.47
Head and neck (open mask)	Intrafractional	Daily	–	–	–	–	–	–	–	–	–	0.0	0.0	0.0	0.0	0.0	0.1	0.0	0.0	0.0	–
Thorax	Intrafractional	Daily	–	–	–	–	–	–	–	–	–	0.0	0.6	1.0	0.1	0.7	1.2	0.1	0.4	0.7	–
Abdomen	Intrafractional	Daily	–	–	–	–	–	–	–	–	–	0.0	0.4	0.9	0.2	0.9	1.5	0.7	0.7	0.8	–
Pelvis	Intrafractional	Daily	–	–	–	–	–	–	–	–	–	− 0.1	0.2	0.6	− 0.1	0.6	1.5	0.5	0.5	1.0	–

*SGRT* Surface guided radiotherapy; *LAT* Lateral; *LNG* Longitudinal; *VRT* Vertical; *M* Mean population error; *Σ* Population systematic error; *σ* Population random error; *p* value Deviation of the three-dimensional vector of the patient setup accuracy using skin marks versus OSMS (Student's *t* test).

### Intra-fractional setup error

The intra-fractional setup error was assessed using 819 radiotherapy fractions of 69 patients positioned using OSMS. The intra-fractional population systematic and random errors were smaller than the corresponding inter-fractional errors on average by a factor of 1.9 (Fig. 2, tab 2). An ANOVA showed no statistically significant impact of the examined factors target volume, fractionation regimen, gender, age, weight, and BMI on the intra-fractional setup error. The mean three-dimensional vector of the intra-fractional setup error of the thorax was 0.9 mm (standard deviation (SD) 0.5 mm), abdomen 0.9 mm (SD 0.3 mm), and pelvis 0.8 mm (SD 0.4 mm).

### Rotational setup error

**Figure 3:**
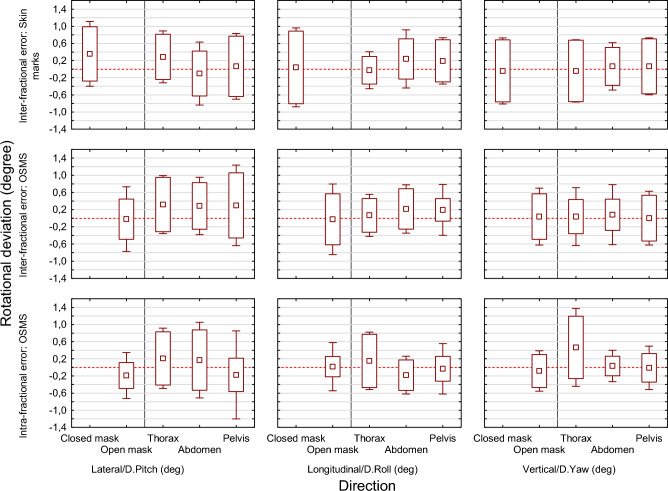
Overall population mean setup error (rectangle), population systematic error (box), and population random error (whiskers) of the inter-fractional rotational error using laser alignment with skin marks, inter-fractional rotational error (mm) using OSMS, and intra-fractional rotational error using OSMS by direction and location of the target volume.

The inter- and intra-fractional rotational errors were analyzed using the same concept of overall population mean setup error, population systematic error and population random error as the translational setup errors. Figure 3 shows that the rotational errors were in the same range in all tumor locations and directions. The mean absolute rotational setup error of the thorax, abdomen, and pelvis in the lateral, longitudinal, and vertical direction was 0.4° (SD 0.4°, range 0.0° to 1.3°) for the inter-fractional error group, and 0.4° (SD 0.5°, range 0.0° to 0.7°) for the intra-fractional error group.

### Calculated safety margin

The calculated setup margins decreased with increasing frequency of online verifications using kV-CBCT (Table 3). Patients positioned using AlignRT required smaller setup margins compared to patients positioned using laser alignment with skin marks. For patients with target volumes in the thorax, abdomen, and pelvis the mean setup margin of the lateral, longitudinal and vertical direction was smaller on average by a factor of 1.3, corresponding to a decrease of the setup margin for the combined inter-and intra-fractional deviation on average by 1.9 mm without online verification using kV-CBCT, 1.3 mm for online verification once per week and 0.9 mm for online verification every other day. For example, patients positioned using laser alignment with skin marks and online verification every other day required similar setup margins as patients positioned using AlignRT and online verification once a week (mean setup margin for the combined inter-and intra-fractional deviation 3.6 mm vs. 3.7 mm) (Table 3).

**Table 3:** Calculated setup margins in dependence of the setup procedure for each scenario.

Setup type		Skin marks						OSMS			Skin marks			OSMS		
Error type		Inter-fractional error (mm)						Intra-fractional error (mm)			Combined inter- and intra-fractional error (mm)					
Anatomical region of the target volume	Frequency of online verifications using kV-CBCT	LAT	LNG	VRT	LAT	LNG	VRT	LAT	LNG	VRT	LAT	LNG	VRT	LAT	LNG	VRT
Head and neck (closed mask)	None	3	3	2	–	–	–	–	–	–	–	–	–	–	–	–
Head and neck (open mask)	None	–	–	–	2	2	2	1	1	1	–	–	–	2	3	2
Thorax	None	4	6	6	3	5	4	2	2	1	5	6	6	3	5	4
Abdomen	None	4	7	7	2	5	3	1	3	2	5	8	7	3	6	3
Pelvis	None	6	6	3	2	6	3	1	2	1	6	6	4	3	7	4
Mean setup margin (thorax/abdomen/pelvis)	None	5	6	5	3	5	3	1	2	1	5	7	6	3	6	4
Mean setup margin (LAT/LNG/VRT)	None		5.5			3.6			1.6			5.9			4.2	
Head and neck (closed mask)	Once per week	2	2	2	–	–	–	–	–	–	–	–	–	–	–	–
Head and neck (open mask)	Once per week	–	–	–	1	2	1	1	1	1	–	–	–	2	2	2
Thorax	Once per week	4	5	5	2	4	3	2	2	1	4	6	5	3	5	3
Abdomen	Once per week	3	5	5	2	4	2	1	3	2	4	6	6	2	5	3
Pelvis	Once per week	4	4	3	2	5	2	1	2	1	5	5	3	2	6	3
Mean setup margin (thorax/abdomen/pelvis)	Once per week	4	5	4	2	4	3	1	2	1	4	6	5	3	5	3
Mean setup margin (LAT/LNG/VRT)	Once per week		4.3			3.0			1.6			4.8			3.7	
Head and neck (closed mask)	Every other day	1	1	1	–	–	–	–	–	–	–	–	–	–	–	–
Head and neck (open mask)	Every other day	–	–	–	1	1	1	1	1	1	–	–	–	1	2	1
Thorax	Every other day	2	3	3	2	2	2	2	2	1	3	4	4	3	3	2
Abdomen	Every other day	2	3	3	2	3	2	1	3	2	3	5	4	2	4	3
Pelvis	Every other day	5	3	1	1	2	2	1	2	1	5	4	2	2	3	2
Mean setup margin (thorax/abdomen/pelvis)	Every other day	3	3	3	2	3	2	1	2	1	3	4	3	2	4	2
Mean setup margin (LAT/LNG/VRT)	Every other day		2.9			2.0			1.6			3.6			2.8	

*OSMS* Optical surface monitoring system; *LAT* Lateral; *LNG* Longitudinal; *VRT* Vertical.

## Discussion

Our data show that for target volumes located in the thorax, abdomen, and pelvis the patient setup accuracy is significantly better using OSMS compared to laser alignment with patient skin marks. Depending on the frequency of online verifications using CBCT, the calculated setup margins to compensate for the combined inter- and intra-fractional setup errors are smaller on average by 1.7 mm (no online verification using CBCT), 1.2 mm (CBCT once a week) or 0.8 mm (CBCT every other day). Furthermore, the intra-fractional setup error can be verified in real time using SGRT which is not possible using CBCT. The mean calculated setup margin in our study to compensate for the intra-fractional error of target volumes located in the thorax, abdomen and pelvis is 1.6 mm.

A major advantage of SGRT for target volumes in the thorax, abdomen and pelvis is the ability to reduce the frequency of CBCTs while maintaining sufficient patient positioning accuracy where clinically appropriate. Reducing the frequency of CBCT scans reduces imaging dose and overall treatment time^3^. Our data show that daily OSMS and CBCT once per week require similar setup margins compared to laser alignment with skin marks and CBCT every other day.

### Inter-fractional setup error

In agreement with our study, significantly smaller inter-fractional setup errors for target volumes of the CNS, head and neck, thorax and abdomen using AlignRT compared to laser alignment with skin marks were observed in a retrospective analysis of 16,835 treatment fractions of 696 patients^3^. The patients were treated using TomoTherapy. Megavoltage computed tomography (MVCT) scans were used as reference for the patient setup accuracy. While the inter-fractional systematic error for target volumes of the thorax and abdomen compared to our study was greater on average by a factor of almost two, the relative reduction of the inter-fractional systematic error using AlignRT was in the same range of our study (factor 0.7). An analysis of 335 fractions of 71 patients treated using SBRT and AlignRT for malignant thoracic or abdominal tumors showed that inter-fractional setup errors were small after SGRT setup (on average < 5 mm and < 0.5°) suggesting that AlignRT may replace skin marks^14^. A study using SBRT for target volumes of the lung, liver, spine, pancreas, and lymph nodes analyzing 284 radiotherapy fractions of 63 patients revealed significantly smaller inter-fractional setup errors using AlignRT compared to laser alignment with skin marks^15^. The absolute median inter-fractional setup deviation was reduced on average by a factor of 0.7 using AlignRT compared to laser alignment with skin marks. Significantly smaller inter-fractional setup errors using AlignRT compared to laser alignment with skin marks for target volumes in the pelvis/lower extremities, abdomen, chest/upper extremities, and breast were found by a retrospective analysis of 6000 individual fractions^16^. The reported average magnitudes of the three-dimensional shift vectors using laser alignment with skin marks were larger on average by a factor of about two compared to our study, and AlignRT reduced the intra-fractional setup error on average by a factor of 0.5. Comparable inter-fractional setup accuracy between AlignRT and laser alignment with skin marks was reported by two study groups analyzing 1902 radiation fractions of 110 patients with target volumes in the head, thorax, abdomen, and extremities^17^ and 154 radiotherapy fractions of 25 patients with target volumes of the thorax, abdomen and pelvis^18^.

### Intra-fractional steup error

Data concerning the intra-fractional setup error using OSMS for target volumes in the thorax (excluding breast), abdomen and pelvis are scarce in the literature. In a study of 335 fractions of 71 patients treated with SBRT for tumors located in the thorax and abdomen in about 10% of the total fractions the intra-fractional setup error exceeded the predefined threshold of 2 mm. The resulting shifts were performed prior to continuation of the treatment. The mean three-dimensional vector of the intra-fractional setup error was 3.3 mm^14^. In an analysis of 792 fractions of 29 patients with target volumes in the pelvis, the mean three-dimensional vector of the intra-fractional setup error was 1.9 mm^19^.

For breast cancer radiotherapy following breast conserving surgery or mastectomy in free breathing position using AlignRT, a study of 2028 radiotherapy fractions of 104 breast cancer patients revealed a median three-dimensional vector of the intra-fractional setup error during dose application of 1.6 mm^20^, and another study of 99 fractions of 10 patients a corresponding intra-fractional setup error of 1.1 mm. The median intra-fractional rotational setup error was 0.4°^21^.

For comparison, the mean three-dimensional vector of the intra-fractional setup errors in our study was 0.9 mm for target volumes of the thorax, 1.1 mm of the abdomen and 0.8 mm of the pelvis.

### Open-face thermoplastic masks

Our data suggest that open-face thermoplastic masks combined with AlignRT provide similar inter-fractional setup accuracy compared to closed-face thermoplastic masks and laser alignment to mask marks. Open-face thermoplastic masks are more comfortable for many patients. However, the intra-fractional error may be increased using open-face masks compared to closed-face masked. In a study of 415 fractions of 269 patients treated with cranial stereotactic radiosurgery (SRS) using open-face mask and AlignRT, an inter-fractional translational setup error of 1.0 mm (SD 2.5 mm) and intra-fractional rotational error of 0.1 degree (SD 1.4 degree) was detected. The authors concluded that SGRT has sufficient accuracy to guide radiotherapy of brain and nasopharynx cancer with standard fractionation^22^. However, given the low setup error when using open-face masks, SGRT in the head and neck region is more relevant when higher doses need to be administered.

### Limitations of the study

Limitations of our study include the general limitations of a retrospective and single-institutional study design. Furthermore, an assumption for the calculation of the population systematic and random error is a comparable number of radiotherapy fractions per patient. In our study, 64% of the patients were treated with hypofractionation (usually 15 fractions), 22% with conventional fractionation (usually between 30 and 35 fractions), and 14% with SBRT (usually five fractions). In addition, during a few radiation fractions, intra-fractional deviations of > 5 mm were observed and a manual beam shut-off performed before setup correction. Due to the relatively large total number of radiation fractions and target volumes examined it is expected that the corresponding statistical errors are small.

## Conclusion

Our data show that for target volumes of the thorax, abdomen, and pelvis the patient setup accuracy using OSMS is significantly better compared to laser alignment with skin marks. The use of OSMS opens the possibility to reduce the number of CBCTs compared to laser alignment with skin marks while keeping the setup accuracy at a sufficient level. Reducing the frequency of CBCT scans reduces imaging dose and overall treatment time. Calculation of the required inter- and intra-fractional setup margins showed that OSMS and once weekly CBCT requires similar setup margins compared to laser alignment with skin marks and CBCT every other day. Open-face thermoplastic masks may be used instead of closed-face thermoplastic mask to increase the patient's comfort.

## Data Availability

The datasets used and/or analysed during the current study are available from the corresponding author on reasonable request.
